# The Human-Specific STING Agonist G10 Activates Type I Interferon and the NLRP3 Inflammasome in Porcine Cells

**DOI:** 10.3389/fimmu.2020.575818

**Published:** 2020-09-24

**Authors:** Sheng-Li Ming, Lei Zeng, Yu-Kun Guo, Shuang Zhang, Guo-Li Li, Ying-Xian Ma, Yun-Yun Zhai, Wen-Ru Chang, Le Yang, Jiang Wang, Guo-Yu Yang, Bei-Bei Chu

**Affiliations:** College of Veterinary Medicine, Henan Agricultural University, Zhengzhou, China

**Keywords:** STING agonist, NLRP3 inflammasome, interferon, negative regulation, innate immunity

## Abstract

Pigs have anatomical and physiological characteristics comparable to those in humans and, therefore, are a favorable model for immune function research. Interferons (IFNs) and inflammasomes have essential roles in the innate immune system. Here, we report that G10, a human-specific agonist of stimulator of interferon genes (STING), activates both type I IFN and the canonical NLRP3 inflammasome in a STING-dependent manner in porcine cells. Without a priming signal, G10 alone transcriptionally stimulated Sp1-dependent *p65* expression, thus triggering activation of the nuclear factor-κB (NF-κB) signaling pathway and thereby priming inflammasome activation. G10 was also found to induce potassium efflux- and NLRP3/ASC/Caspase-1-dependent secretion of IL-1β and IL-18. Pharmacological and genetic inhibition of NLRP3 inflammasomes increased G10-induced type I IFN expression, thereby preventing virus infection, suggesting negative regulation of the NLRP3 inflammasome in the IFN response in the context of STING-mediated innate immune activation. Overall, our findings reveal a new mechanism through which G10 activates the NLRP3 inflammasome in porcine cells and provide new insights into STING-mediated innate immunity in pigs compared with humans.

## Introduction

The innate immune system detects pathogen-associated molecular patterns (PAMPs) or danger-associated molecular patterns (DAMPs) *via* germline-encoded pattern recognition receptors (PRRs) (1). Subsequently, innate immune responses are activated, and inflammatory cytokines, such as interferons (IFNs), proinflammatory cytokines, and chemokines, are generated. DAMPs and PAMPs comprise self- and foreign-derived double-stranded DNA in the cytosol (2). Stimulator of interferon genes (STING) is an ER-resident adaptor protein that is critical in mediating the signaling triggered by cytosolic nucleic acids (3, 4). After activation by an agonist, STING undergoes a conformational change resulting in the recruitment of TANK binding kinase (TBK1) to STING (5, 6). TBK1 subsequently phosphorylates IFN-regulated factor 3 (IRF3) and nuclear factor-κB (NF-κB), which translocate into the nucleus and stimulate expression of type I IFN and proinflammatory cytokines (7). Given the importance of the STING-mediated pathway in the activation of innate immunity and host protection from pathogens, harnessing the innate immunity activated by STING agonists is a promising strategy for antiviral and antitumor therapeutics (8, 9). G10 is a synthetic small molecule that indirectly activates human STING and triggers IRF3-dependent IFNs expression but not NF-κB activation, thereby protecting against infection with emerging alphaviruses (10).

Inflammasomes are intracellular supramolecular complexes that assemble in response to the detection of microbial infection or stress-associated stimuli in innate immunity. The assembly of inflammasomes is a well-regulated process initiated by the recognition of DAMPs and PAMPs by PRRs (11). The nucleotide-binding domain, leucine-rich-repeat-containing proteins (NLRs), including NLRP1, NLRP3, NLRP6, NLRP7, and NLRP9, are notable inflammasome-forming PRRs (12–17). The NLRP3 inflammasome, the best-characterized inflammasome, contains NLRP3, the adaptor protein apoptosis-associated speck-like protein containing a caspase-recruitment domain (ASC) and the proinflammatory protein Caspase-1 (18). Activation of the NLRP3 inflammasome requires a priming signal and an activating signal. The priming process often involves TLRs, which activate NF-κB, thus resulting in the expression and activation of NLRP3, pro–IL-1β, and pro–IL-18 (19). Canonical activation is characterized by the oligomerization of NLRP3, ASC, and pro–Caspase-1, thus leading to the maturation of the proinflammatory cytokines IL-1β and IL-18, and the induction of pyroptotic cell death (20). The NLRP3 inflammasome is activated after exposure to a broad range of signals, including potassium efflux, calcium mobilization, mitochondrial damage, and reactive oxygen species (ROS) (21–24).

Activation of innate immunity by DAMPs and PAMPs usually leads to type I IFN expression and inflammasome activation. Because IL-1β, IL-18, and type I IFN are key players in both infectious and autoimmune diseases, reciprocal regulation between IFNs and inflammasome is essential for immune homeostasis. Type I IFN has been reported to induce Caspase-11 expression, thereby activating non-canonical inflammasome (25), whereas other studies have suggested that type I IFN inhibits inflammasome activation (26). IFN-inducible PYRIN domain (PYD)-only protein 3 interferes with the interaction between absent in melanoma 2 (AIM2) and ASC, thus inhibiting the AIM2 inflammasome (27). Another IFN-inducible protein, cholesterol 25-hydroxylase, converts cholesterol into 25-hydroxycholesterol, thus inhibiting pro–L-1β transcription and inflammasome activation (28). In contrast, type I IFN is antagonized by inflammasomes (29, 30). Caspase-1 cleaves cyclic GMP-AMP synthase (cGAS), thus inhibiting cGAS-STING-mediated type I IFN production (31).

Pigs are a validated model for use in biomedical research fields, such as xenotransplantation and immune disorders (32, 33). However, the interplay between type I IFN and inflammasomes has not been well documented in pigs to date. Here, we report that G10 triggers the activation of a STING-dependent type I IFN response and the NLRP3 inflammasome in porcine cells. G10 provides priming and activating signals, both of which are required for NLRP3 inflammasome activation. Furthermore, the NLRP3 inflammasome negatively regulates the type I IFN response in porcine cells after G10 treatment. Our results reveal a new mechanism through which G10 activates the NLRP3 inflammasome in porcine cells.

## Materials and Methods

### Antibodies and Reagents

The antibodies anti-P65 (#8242), anti-p-P65 (#3033), and anti-Lamin B1 (#13435) were from Cell Signaling Technology; anti-Flag M2 (F1804) was from Sigma; anti-Sp1 (10500-1-AP), anti-ASC (21962-1-AP), and anti-β-actin (20536-1-AP) were from Proteintech; anti-IL-1β was from R&D Systems (AF-401-NA); anti-Caspase-1 was from Santa Cruz Biotechnology (sc-398715).

G10 (HY-19711), LPS (HY-D1056), ATP (HY-B2176), nigericin (HY-127019), MCC950 (HY-12815), and VX765 (HY-13205) were from MedChemExpress; and CL097 (tlrl-c97) was from InvivoGen.

### Cells and Viruses

PK-15 (CCL-33, ATCC), 3D4/21 (CRL-2843, ATCC), HEK293 (CRL-1573, ATCC), HEK293T (CRL-11268, ATCC), and Vero (CL-81, ATCC) were cultured in DMEM (10566-016, GIBCO) supplemented with 10% FBS (10099141C, GIBCO), 100 units/ml penicillin, and 100 μg/ml streptomycin sulfate (B540732, Sangon). THP-1 (TIB-202, ATCC) were cultured in RPMI 1640 (21870-076, GIBCO) supplemented with 10% FBS, 100 units/ml penicillin, and 100 μg/ml streptomycin sulfate. All cells were grown in monolayers at 37°C under 5% CO_2_. PRV-QXX was used as previously described (34).

### THP-1 Macrophage Differentiation

THP-1 cells were differentiated overnight with DMEM/10% FBS supplemented with 100 ng/ml phorbol-12-myristate 13-acetate (P1585, Sigma). After being washed three times with PBS, the cells were re-plated in DMEM/10% FBS and allowed to recover for 2 days.

### Cell Stimulation

For NLRP3 inflammasome activation, cells were primed by incubation with LPS (1 μg/ml) at 37°C for 3 h. Then, NLRP3 inflammasomes were activated by incubation of cells with nigericin (2.5 μM, denoted LPS + Nig) or ATP (5 mM, denoted LPS + ATP) for the indicated times.

### Plasmids

The plasmids pcDNA3-N-Flag-NLRP3 (#75127) and pLEX-MCS-ASC-GFP (#73957) were from Addgene. The human STING-Flag plasmid was a gift from Chun-Fu Zheng (Fujian Medical University, China).

To clone individual small guide RNAs (sgRNAs), 24-bp oligonucleotides containing the sgRNA sequences were synthesized (Sangon). These sequences included a 4-bp overhang in the forward (CACC) and complementary reverse (AAAC) oligonucleotides to enable cloning into the *Bsm*BI site of the lentiCRISPR v2 (#52961, Addgene). The sgRNA sequences were as follows: *p65*-sgRNA1: 5′-GCGCTCAGCCGGCAGTATCC-3′; *p65*-sgRNA2: 5′-GCTCTCGCCCGGGATACTGC-3′; *Nlrp3*-sgRNA: 5′-GTGCAAGCTGGCTCGTTACC-3′; *Asc*-sgRNA: 5′-GAAGCTCGTCAACTACTACC-3′; and *Caspase-1*-sgRNA: 5′-GAACGCTACAGTTATGGATA-3′. The sgRNA sequences for *Sting*, *Tbk1*, *Irf3*, and *Ifnar1*were used as previously described (34).

For luciferase reporter assays, the porcine *p65* promoter 1800 bp upstream of the transcription initiation site (+1), as well as Sp1 binding site mutants, were synthesized (Genscript) and cloned into pGL3-Basic (E1751, Promega) through the *Kpn*I and *Hind*III sites to generate *p65*-Luc plasmids. Sp1 binding site 1 (−698) of 5′-CCGGCAGTGA-3′ was mutated to 5′-ACAAAAAAAC-3′ (*p65*-Luc mut1). Sp1 binding site 2 (−1300) of 5′-GAGGCACGGA-3′ was mutated to 5′-ACAAACAAAC-3′ (*p65*-Luc mut2). Sp1 binding site 3 (−482) of 5′-CCGGCAGTGA-3′ was mutated to 5′-AAAAACAGAC-3′ (*p65*-Luc mut3).

### RT-qPCR

Total RNA was extracted from cells with TRIzol Reagent (9108, TaKaRa) and reverse-transcribed to cDNA with a PrimeScript RT reagent Kit (RR047A, TaKaRa) according to the manufacturer’s instructions. RT-qPCR was performed in triplicate with SYBR Premix Ex Taq (RR820A, TaKaRa) according to the manufacturer’s instructions. The results were normalized to the level of β-actin expression. Amounts were quantified with the 2^–ΔΔCt^ method. Primer sequences are as follows: porcine IFN-β-Fw: 5′-AGTTGCCTGGGACTCCTCAA-3′, porcine IFN-β-Rv: 5′-CCTCAGGGACCTCAAAGTTCAT-3′; porcine IL-1β-Fw: 5′-GCCCTGTACCCCAACTGGTA-3′, porcine IL-1β-Rv: 5′-CCAGGAAGACGGGCTTTTG-3′; porcine IL-18-Fw: 5′-AGGGACATCAAGCCGTGTTT-3′, porcine IL-18- Rv: 5′-CGGTCTGAGGTGCATTATCTGA-3′; porcine P65-Fw: 5′-GCATCCACAGCTTCCAGAAC-3′, porcine P65-Rv: 5′-GC ACAGCATTCAGGTCGTAG-3′; porcine ISG15-Fw: 5′-AAGG TGAAGATGCTGGGAGG-3′, porcine ISG15-Rv: 5′-CAGGATG CTCAGTGGGTCTC-3′; porcine NLRP1-Fw: 5′-TGAGTGAGG AGCAGTATGA-3′, porcine NLRP1-Rv: 5′-CAGAGCAGGTGT TCAGAC-3′; porcine NLRP3-Fw: 5′-AGAGGAGGAGGAGG AAGA-3′, porcine NLRP3-Rv: 5′-CACCAATCGCTGAGAATAT G-3′; porcine β-actin-Fw: 5′-CTGAACCCCAAAGCCAACCGT- 3′, porcine β-actin-Rv: 5′-TTCTCCTTGATGTCCCGCACG-3′.

### Immunoblotting Analysis

Cells were washed once in PBS and lysed with cell lysis buffer (50 mM Tris–HCl, pH 8.0, 150 mM NaCl, 1% Triton X-100, 1% sodium deoxycholate, 0.1% SDS, and 2 mM MgCl_2_) supplemented with protease and phosphatase inhibitor cocktails (HY-K0010 and HY-K0022, MedChemExpress). Lysates were then centrifuged at 13,000 rpm for 15 min, and the insoluble fraction was removed. Protein concentrations were measured with a BCA Protein Assay Kit (BCA01, DINGGUO Biotechnology). Equal amounts of protein were loaded onto each lane and separated by SDS-PAGE, transferred to nitrocellulose membranes (ISEQ00010, Millipore), and incubated in 5% nonfat milk (A600669, Sangon) for 1 h at room temperature. The membranes were incubated with primary antibody at 4°C overnight and then incubated with horseradish-peroxidase-conjugated secondary antibody (709-035-149 or 715-035-150, Jackson ImmunoResearch Laboratories) for 1 h. Immunoblotting results were visualized with Luminata Crescendo Western HRP Substrate (WBLUR0500, Millipore) on a GE AI600 imaging system.

### Nuclear and Cytoplasmic Extraction

Nuclear and cytoplasmic extraction was performed with NE-PER Nuclear and Cytoplasmic Extraction Reagents (78833, Thermo Fisher Scientific) according to the manufacturer’s instructions. The extracted fractions were subjected to immunoblotting analysis.

### ASC Speck Oligomerization Assay

Cells were lysed in homogenization buffer (20 mM HEPES-KOH, pH 7.5, 10 mM KCl, 1.5 mM MgCl_2_, 1 mM EDTA, 1 mM EGTA, and 320 mM sucrose) supplemented with protease inhibitor cocktail via passage through a 21-gage needle 30 times. The lysates were subjected to centrifugation at 1500 rpm for 10 min. The supernatants were diluted with 1 volume of CHAPS buffer (20 mM HEPES-KOH, pH 7.5, 5 mM MgCl_2_, 0.5 mM EGTA, and 0.1% CHAPS) supplemented with protease inhibitor cocktail and were centrifuged at 5000 rpm for 10 min. The pellets were washed three times with ice-cold PBS and then re-suspended in 30 μl of CHAPS buffer supplemented with 2 mM DSS crosslinker (21655, Thermo Fisher Scientific). After incubation at 37°C for 20 min, the reaction was quenched by the addition of Laemmli buffer, and the samples were subjected to SDS-PAGE.

### Immunofluorescence Analysis

Cells were grown on coverslips (12-545-80, Thermo Fisher Scientific) in 12-well plates and fixed with PBS/4% paraformaldehyde at room temperature for 30 min. The cells were then permeabilized with PBS/0.1% Triton X-100 at room temperature for 3 min. After being washed twice with PBS, the cells were incubated with PBS/10% FBS supplemented with the primary antibody at room temperature for 1 h. After being washed three times with PBS, the cells were labeled with 10% FBS/PBS supplemented with fluorescent secondary antibody (#A-11034, Thermo Fisher Scientific) at room temperature for 1 h. Images were captured under a Zeiss LSM 800 confocal microscope and processed in ImageJ software for quantitative image analysis.

### Dual Luciferase Reporter Assays

Cells cultured in 24-well plates were co-transfected with pCMV–Renilla (normalization plasmid) and *p65*–Luc (luciferase reporter plasmid) with Lipofectamine 3000 (L3000015, Invitrogen). At 24 h post transfection, luciferase reporter assays were performed with the Dual-Luciferase Reporter Assay System (E1910, Promega) according to the manufacturer’s instructions. The luminescence signal was detected with a Fluoroskan Ascent FL Microplate Fluorometer (Thermo Fisher Scientific).

### Chromatin Immunoprecipitation Assays

Cells grown in 10-cm dishes were cross-linked with DMEM containing 1% formaldehyde for 15 min, and then the crosslinking was stopped by the addition of 125 mM glycine for 5 min. After being washed twice with PBS, the cells were incubated in lysis buffer (10 mM Tris–HCl, pH 7.5, 10 mM KCl, 5 mM MgCl_2_, and 0.5% NP40) supplemented with protease inhibitor cocktail on ice for 10 min and centrifuged at 2000 rpm for 5 min. The cell pellets containing chromatin were suspended in SDS lysis buffer (50 mM Tris–HCl, pH 7.9, 10 mM EDTA, and 0.5% SDS) supplemented with protease inhibitor cocktail and sonicated into fragments with an average length of 1 kb. Chromatin immunoprecipitation (ChIP) assays were performed with IgG or antibody against Sp1. Primer specific for *p65* promoter was as follows: Fw: 5′-CCCCTCGGTGCCTTCT-3′ and Rv: 5′-CGATGGGTGCACGCTA-3′.

### Lentivirus Production

HEK293T cells were seeded at 4 × 10^6^ cells per 10-cm dish in DMEM/10% FBS. Cells were cultured for 24 h and transfected with 2 μg/dish lentiviral construct, 1.5 μg/dish psPAX2 (packaging plasmid, #12260, Addgene), and 0.5 μg/dish pMD2.G (envelope plasmid, #12259, Addgene) with Lipofectamine 3000 (Invitrogen). After 18 h, the medium was aspirated and replaced with 10 ml of DMEM/10% FBS. Lentivirus particles were harvested at 48 and 72 h post transfection and stored at −80°C.

### Generation of Gene Knockout Cell Lines via CRISPR/Cas9

*Sting*^–/–^, *Tbk1*^–/–^, *Irf3*^–/–^, and *Ifnar1*^–/–^ PK15 cells were used as previously described (34). *Sting*^–/–^ and *Ifnar1*^–/–^ 3D4/21 cells were generated through the same procedure as *Sting*^–/–^ and *Ifnar1*^–/–^ PK15 cells (34). *p65*^–/–^, *Nlrp3*^–/–^, *Asc*^–/–^, and *Caspase-1*^–/–^ PK15 cells and *p65*^–/–^, *Nlrp3*^–/–^ and *Asc*^–/–^, *Caspase-1*^–/–^ 3D4/21 cells were generated by infection of the cells with lentivirus particles containing SpCas9 and the sgRNAs of interest for 48 h and then selected with puromycin (4 μg/ml) for 7 days. Single clonal knockout cells were obtained by serial dilution and verified by Sanger sequencing.

### RNA Interference

Cells were seeded in 60-mm dishes at a density of 4 × 10^5^ cells per dish and were transfected with small interfering RNA (siRNA, GenePharma, Shanghai) at a final concentration of 0.12 nM. Transfections were performed with Lipofectamine RNAiMAX Reagent (13778500, Invitrogen) according to the manufacturer’s instructions in Opti-MEM reduced serum medium (31985062, GIBCO). The medium was replaced with DMEM containing 10% FBS at 8 h post-transfection. The knockdown efficacy was assessed by immunoblotting analysis at 48 h post-transfection. The siRNA sequences were as follows: siControl: 5′-UUCUCCGAACGUGUCACGU-3′; siSp1–1: 5′-GCAACAUCAUUGCUGCUAU-3′; siSp1–2: 5′-GGGAAACGCUUCACACGUU-3′; and siSp1–3: 5′-CCAUUAAUAUCAGUGGCAA-3′.

### ELISA

Concentrations of IFN-β and interleukins were measured in the cell supernatants with ELISA kits from R&D Systems (human IL-1β, PDLB50 and porcine IL-1β, PLB00B) and Advanced BioChemical (porcine IFN-β, ABCE-EL-P1819 and porcine IL-18, ABCE-EL-P007), according to the manufacturers’ instructions.

### Caspase-1 Activity Assays

Caspase-1 activity was assessed with a Caspase-Glo 1 Inflammasome Assay kit (G9951, Promega) with cell lysates treated as indicated, according to the manufacturer’s instructions.

### The Tissue Culture Infective Dose Assays

Vero cells were seeded in 96-well plates at a density of 1 × 10^4^ cells per well. On the next day, the cells were inoculated with serially diluted viruses (10^–1^–10^–12^-fold) for 1 h at 37°C. The excess viral inoculum was removed by washing with PBS. Then, 200 μl of DMEM/2% FBS was added to each well, and the cells were further cultured for 3–5 days. The cells demonstrating the expected cytopathic effects were observed daily, and the tissue culture infective dose (TCID_50_) value was calculated with the Reed–Muench method.

### Plaque Assays

Vero cells were cultured just to confluency in six-well plates and inoculated with serially diluted viruses (10^–1^–10^–7^-fold) for 1 h at 37°C. The excess viral inoculum was removed by washing with PBS. Then, 4 ml of DMED/1% methylcellulose (M8070, Solarbio) was added to each well, and the cells were further cultured for 4–5 days. The cells were fixed with 4% paraformaldehyde for 15 min and stained with 1% crystal violet for 30 min before the plaques were counted.

### Statistical Analysis

GraphPad Prism 7 software was used for data analysis. Data are shown as mean ± standard deviations from three independent experiments. Statistical significance between two groups was analyzed with two-tailed unpaired Student’s *t*-test or one-way ANOVA.

## Results

### G10 Activates the Type I IFN Response in Porcine Cells

**FIGURE 1:**
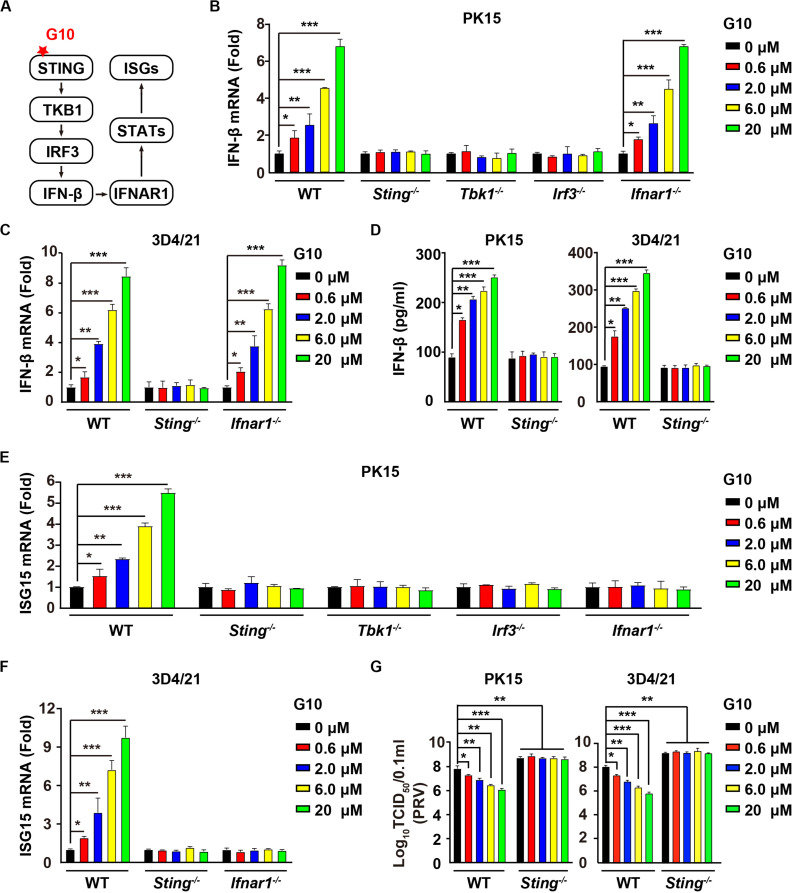
G10 elicits a type I IFN response in porcine cells. **(A)** Schematic representation of the STING-mediated type I IFN response. **(B)** WT, *Sting*^–/–^, *Tbk1*^–/–^, *Irf3*^–/–^, and *Ifnar1*^–/–^ PK15 cells were seeded in 12-well plates at a density of 1 × 10^5^ per well. On the next day, cells were treated with G10 at the indicated concentrations for 24 h. Total mRNA was then reverse-transcribed to cDNA and IFN-β mRNA was assessed by RT-qPCR analysis. The results were normalized to the level of β-actin expression. *P < 0.05, **P < 0.01, ***P < 0.001 determined by two-tailed Student’s *t*-test. **(C)** WT, *Sting*^–/–^, and *Ifnar1*^–/–^ 3D4/21 cells were seeded in 12-well plates at a density of 1 × 10^5^ per well. On the next day, cells were treated as in **B**. IFN-β mRNA was assessed by RT-qPCR analysis. The results were normalized to the level of β-actin expression. *P < 0.05, **P < 0.01, ***P < 0.001 determined by two-tailed Student’s *t*-test. **(D)** WT and *Sting*^–/–^ PK15 and 3D4/21 cells were seeded in 12-well plates at a density of 1 × 10^5^ per well. On the next day, cells were treated as in **B**. The medium was then harvested and IFN-β secretion was quantified by ELISA. *P < 0.05, **P < 0.01, ***P < 0.001 determined by two-tailed Student’s *t*-test. **(E)** WT, *Sting*^–/–^, *Tbk1*^–/–^, *Irf3*^–/–^, and *Ifnar1*^–/–^ PK15 cells were seeded in 12-well plates at a density of 1 × 10^5^ per well. On the next day, cells were treated as in **B**. ISG15 mRNA was assessed by RT-qPCR analysis. The results were normalized to the level of β-actin expression. *P < 0.05, **P < 0.01, ***P < 0.001 determined by two-tailed Student’s *t*-test. **(F)** WT, *Sting*^–/–^, and *Ifnar1*^–/–^ 3D4/21 cells were seeded in 12-well plates at a density of 1 × 10^5^ per well. On the next day, cells were treated as in **B**. ISG15 mRNA was assessed by RT-qPCR analysis. The results were normalized to the level of β-actin expression. *P < 0.05, **P < 0.01, ***P < 0.001 determined by two-tailed Student’s *t*-test. **(G)** WT and *Sting*^–/–^ PK15 and 3D4/21 cells were seeded in 12-well plates at a density of 1 × 10^5^ per well. On the next day, cells were infected with PRV-QXX (MOI = 1) and simultaneously treated with G10 as in **B**. Virus was harvested by three freeze–thaw cycles and PRV titer was assessed with TCID_50_ assays. *P < 0.05, **P < 0.01, ***P < 0.001 determined by one-way ANOVA.

G10 is a human-specific STING agonist that activates the IFN response (Figure 1A) (10). We aimed to determine whether G10 might activate porcine STING-mediated type I IFN activation. We found that G10 up-regulated the transcription of IFN-β mRNA in both porcine PK15 kidney epithelial cells and 3D4/21 alveolar macrophages, in a manner dependent on STING and its downstream effectors TBK1 and IRF3, but not on IFNAR1 (Figures 1B,C). Ablation of *Sting* abolished G10-stimulated IFN-β secretion in PK15 and 3D4/21 cells (Figure 1D). We further detected the expression of IFN-stimulated gene 15 (ISG15) under G10 treatment by using RT-qPCR analysis. As shown in Figure 1E, challenge of *Sting*^–/–^, *Tbk1*^–/–^, *Irf3*^–/–^, and *Ifnar1*^–/–^ PK15 cells with G10 had no effect on *ISG15* transcription. This finding was also confirmed in *Sting*^–/–^ and *Ifnar1*^–/–^ 3D4/21 cells, thus suggesting that G10 activates type I IFN and IFN signaling in porcine cells (Figure 1F).

We next assessed the antiviral activity of G10 against pseudorabies virus (PRV), a member of the subfamily Alphaherpesvirinae in the family Herpesviridae, and the causative pathogen of Aujeszky’s disease in pigs (35). G10 treatment inhibited PRV infection in porcine PK15 and 3D4/21 cells (Figure 1G). STING deficiency in PK15 and 3D4/21 cells abrogated the antiviral activity of G10 and enhanced PRV infection (Figure 1G). These findings demonstrate that G10 acts on STING, resulting in activation of type I IFN and antiviral activity in porcine cells.

### G10 Activates P65 Gene Transcription in Porcine Cells

**FIGURE 2:**
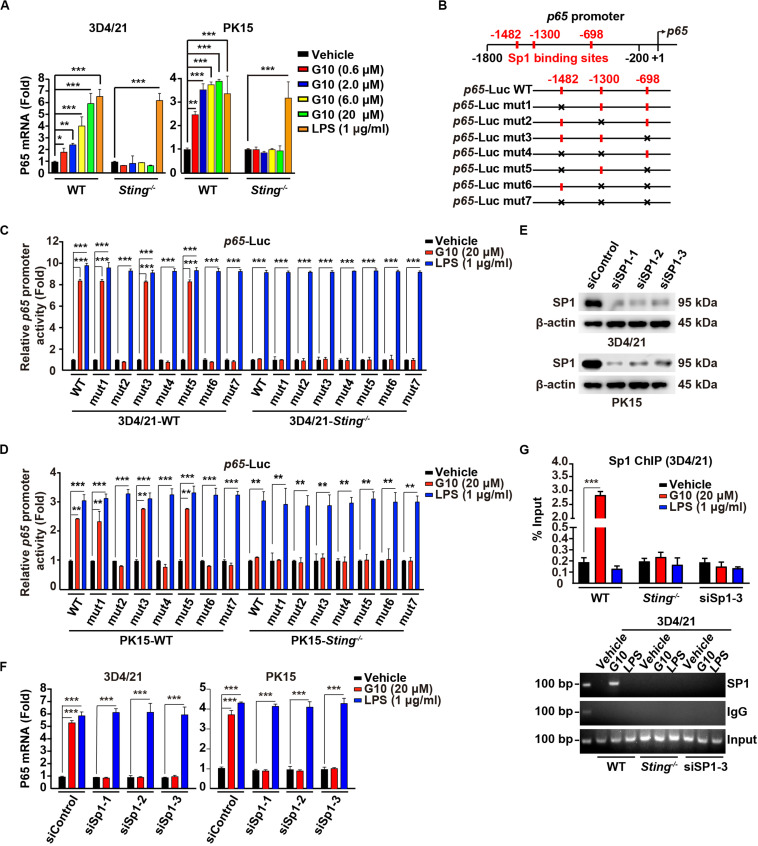
G10 stimulates Sp1-dependent *p65* transcription in porcine cells. **(A)** WT and *Sting*^–/–^ 3D4/21 and PK15 cells were seeded in 12-well plates at a density of 1 × 10^5^ per well. On the next day, cells were treated with vehicle (DMSO), G10 (0.6–20 μM), and LPS (1 μg/ml) for 24 h. Total mRNA was then reverse-transcribed to cDNA and P65 mRNA was assessed by RT-qPCR analysis. The results were normalized to the level of β-actin expression. *P < 0.05, **P < 0.01, ***P < 0.001 determined by two-tailed Student’s *t*-test. **(B)** Diagrams of the P65 promoter and the various mutants, with the Sp1 binding sites indicated. **(C)** WT and *Sting*^–/–^ 3D4/21 cells were seeded in 12-well plates at a density of 1 × 10^5^ per well. On the next day, cells were transfected with 0.1 μg/well *p65*–Luc variants and 0.02 μg/well pCMV-Renilla. At 24 h post transfection, cells were treated with vehicle (DMSO), G10 (20 μM), and LPS (1 μg/ml) for 6 h. *p65* promoter activity was assessed with dual luciferase reporter assays. ***P < 0.001 determined by two-tailed Student’s *t*-test. **(D)** *p65* promoter activity was assessed with dual luciferase reporter assays in WT and *Sting*^–/–^ PK15 cells the same as in C. **P < 0.01, ***P < 0.001 determined by two-tailed Student’s *t*-test. **(E)** 3D4/21 and PK15 cells were seeded in 60-mm dishes at a density of 4 × 10^5^ per dish. On the next day, cells were transfected with indicated siRNA for 48 h. Sp1 protein was assessed with immunoblotting analysis. β-actin served as loading control. **(F)** 3D4/21 and PK15 cells were seeded in 12-well plates at a density of 1 × 10^5^ per well. On the next day, cells were transfected with indicated siRNA for 48 h. Then cells were treated with vehicle (DMSO), G10 (20 μM), and LPS (1 μg/ml) for 24 h. Total mRNA was then reverse-transcribed to cDNA and P65 mRNA was assessed by RT-qPCR analysis. The results were normalized to the level of β-actin expression. ***P < 0.001 determined by two-tailed Student’s *t*-test. **(G)** WT and *Sting*^–/–^ 3D4/21 cells were seeded in 60-mm dishes at a density of 4 × 10^5^ per dish. On the next day, 3D4/21 cells were transfected with siSp1-3 for 48 h. Then WT, *Sting*^–/–^, and siSp1-3 transfected 3D4/21 cells were treated with vehicle (DMSO), G10 (20 μM), and LPS (1 μg/ml). Sp1 ChIP assays were performed at 24 h post treatment. ***P < 0.001 determined by two-tailed Student’s *t*-test.

G10 activates human STING and triggers IRF3-dependent IFNs expression but not NF-κB activation. Unexpectedly, porcine 3D4/21 and PK15 cells showed significant P65 mRNA up-regulation in response to G10 in a dose-dependent manner (Figure 2A). Knockout of STING inhibited G10-, but not LPS-induced *p65* transcription in 3D4/21 and PK15 cells (Figure 2A). Replenishment of STING expression in *Sting*^–/–^ 3D4/21 cells rescued G10-induced *p65* transcription (Supplementary Figure S1A).

We next sought to address how G10 transcriptionally activates porcine *p65* expression. We analyzed the promoter of the porcine *p65* gene and found three consensus binding sites for the transcriptional factor Sp1 (Figure 2B). Promoter mutation analysis with dual luciferase reporter assays indicated that the second Sp1 binding site (−1300) in the *p65* promoter was essential for G10-mediated induction of *p65* expression in a STING-dependent manner in 3D4/21 and PK15 cells (Figures 2C,D). However, LPS-stimulated *p65* transcription was not dependent on these three Sp1 binding sites or on STING (Figures 2C,D). In addition, knockdown of Sp1 with RNA interference affected the expression of P65 mRNA in 3D4/21 and PK15 cells under G10 treatment (Figures 2E,F). Knockdown of Sp1 did not prevent LPS-induced *p65* transcription, thus suggesting that G10 and LPS regulate *p65* transcription through different mechanisms in porcine cells (Figure 2F). Furthermore, ChIP assays showed that Sp1 was recruited to the promoter of *p65* after G10 treatment, in a manner dependent on either STING or Sp1 (Figure 2G). Collectively, these data suggest that G10 induces *p65* transcription through STING and Sp1 in porcine cells.

### G10 Activates the Porcine NF-κB Signaling Pathway

**FIGURE 3:**
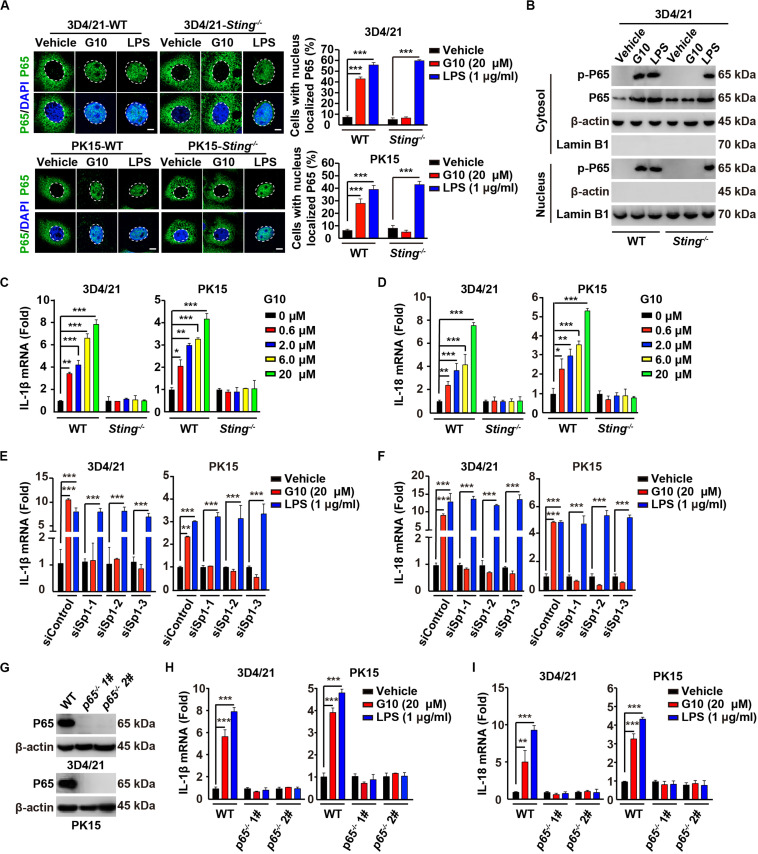
G10 activates the NF-κB signaling pathway in porcine cells. **(A)** WT and *Sting*^–/–^ 3D4/21 and PK15 cells were seeded in 12-well plates with coverslips at a density of 1 × 10^5^ per well. On the next day, cells were treated with vehicle (DMSO), G10 (20 μM), and LPS (1 μg/ml) for 24 h. Translocation of P65 into the nucleus (DAPI) was assessed by immunofluorescence analysis with antibody against P65. Quantification of cells with nuclear localized P65 is shown on the right (*n* = 30 cells). Scale bar, 10 μm. ***P < 0.001 determined by two-tailed Student’s *t*-test. **(B)** WT and *Sting*^–/–^ 3D4/21 cells were seeded in 60-mm dishes at a density of 4 × 10^5^ per dish. On the next day, cells were treated as in **A**. Phospho-P65 and P65 were assessed with immunoblotting analysis in the cytosol and nuclei fraction. β-actin (indicating cytosol) and Lamin B1 (indicating nuclei) served as loading controls. **(C,D)** WT and *Sting*^–/–^ 3D4/21 and PK15 cells were seeded in 12-well plates at a density of 1 × 10^5^ per well. On the next day, cells were treated with G10 at the indicated concentration for 24 h. Total mRNA was then reverse-transcribed to cDNA and IL-1β **(C)** and IL-18 **(D)** mRNAs were assessed by RT-qPCR analysis. The results were normalized to the level of β-actin expression. *P < 0.05, **P < 0.01, ***P < 0.001 determined by two-tailed Student’s *t*-test. **(E,F)** 3D4/21 and PK15 cells were seeded in 12-well plates at a density of 1 × 10^5^ per well. On the next day, cells were transfected with indicated siRNAs for 48 h. Then, cells were treated as in **A**. Total mRNA was then reverse-transcribed to cDNA and IL-1β **(E)** and IL-18 **(F)** mRNAs were assessed by RT-qPCR analysis. The results were normalized to the level of β-actin expression. **P < 0.01, ***P < 0.001 determined by two-tailed Student’s *t*-test. **(G)** P65 protein was assessed with immunoblotting analysis in WT, *p65*^–/–^ 1#, and *p65*^–/–^ 2# 3D4/21 and PK15 cells. β-actin served as loading control. **(H,I)** WT, *p65*^–/–^ 1#, and *p65*^–/–^ 2# 3D4/21 and PK15 cells were seeded in 12-well plates at a density of 1 × 10^5^ per well. On the next day, cells were treated as in **A**. Total mRNA was then reverse-transcribed to cDNA and IL-1β **(H)** and IL-18 **(I)** mRNAs were assessed by RT-qPCR analysis. The results were normalized to the level of β-actin expression. **P < 0.01, ***P < 0.001 determined by two-tailed Student’s *t*-test.

On the basis of the above findings, we attempted to determine whether G10 might activate the porcine NF-κB signaling pathway. We first addressed whether G10 induced the translocation of P65 into the nucleus. G10 did not stimulate P65 translocation into the nucleus, as indicated by immunofluorescence in human THP-1 cells (Supplementary Figure S1B). However, treatment of wild-type (WT) 3D4/21 and PK15 cells with G10 significantly promoted STING-dependent nuclear localization of P65 (Figure 3A). However, the percentage of cells with nuclear localized P65 in WT and *Sting*^–/–^ 3D4/21 and PK15 cells was unchanged by LPS treatment (Figure 3A). LPS, but not G10, induced P65 translocation into the nucleus in *Sting*^–/–^ 3D4/21 cells, as indicated by immunoblotting of nuclear and cytoplasmic extracts (Figure 3B).

We next investigated the transcription of the NF-κB target genes *Il-1*β and *Il-18* (36). G10 treatment significantly induced the transcription of IL-1β and IL-18 mRNA in 3D4/21 and PK15 cells, in a manner dependent on STING (Figures 3C,D). Knockdown of Sp1 abrogated G10- but not LPS-induced transcription of IL-1β and IL-18 mRNA (Figures 3E,F). Furthermore, we ablated *p65* with CRISPR/Cas9 editing in 3D4/21 and PK15 cells (Figure 3G and Supplementary Figure S1C). The mRNA levels of IL-1β and IL-18 were not up-regulated by the treatment of G10 and LPS in *p65*^–/–^ 3D4/21 and PK15 cells, thus suggesting that G10- and LPS-induced expression of IL-1β and IL-18 mRNA was dependent on P65 (Figures 3H,I). These data indicate that G10 activates the NF-κB signaling pathway in porcine cells.

### G10 Induces IL-1β and IL-18 Secretion in Porcine Cells

**FIGURE 4:**
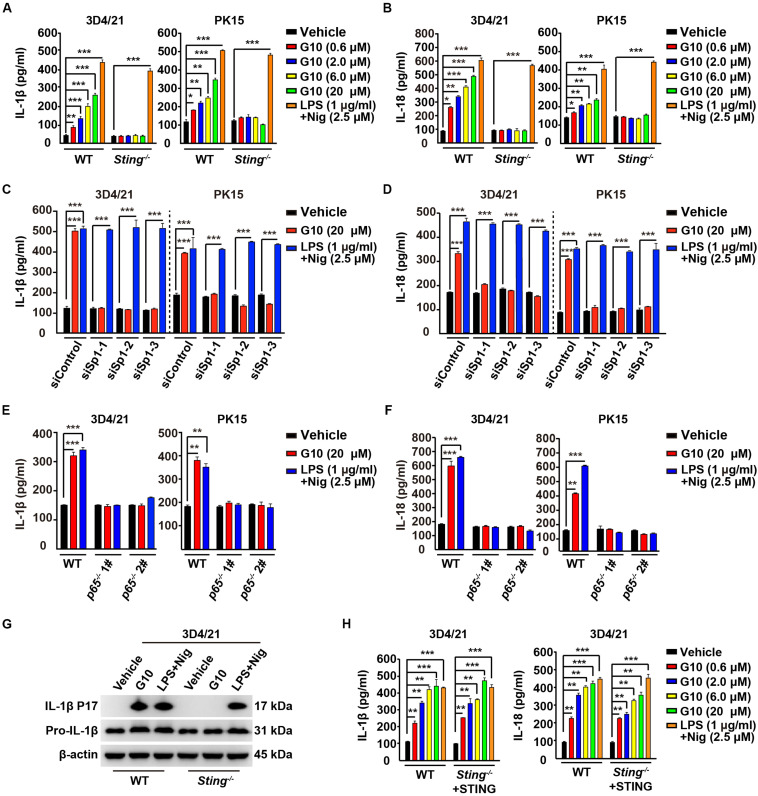
G10 promotes IL-1β and IL-18 secretion in porcine cells. **(A,B)** WT and *Sting*^–/–^ 3D4/21 and PK15 cells were seeded in 12-well plates at a density of 1 × 10^5^ per well. On the next day, cells were treated with vehicle (DMSO), G10, and LPS + Nig at indicated concentrations for 24 h. The medium was then harvested and IL-1β **(A)** and IL-18 **(B)** secretion was quantified by ELISA. *P < 0.05, **P < 0.01, ***P < 0.001 determined by two-tailed Student’s *t*-test. **(C,D)** 3D4/21 and PK15 cells were seeded in 12-well plates at a density of 1 × 10^5^ per well. On the next day, cells were transfected with indicated siRNA for 48 h. Then, cells were treated with vehicle (DMSO), G10, and LPS + Nig at indicated concentrations for 24 h. The medium was then harvested and IL-1β **(C)** and IL-18 **(D)** secretion was quantified by ELISA. ***P < 0.001 determined by two-tailed Student’s *t*-test. **(E,F)** WT, *p65*^–/–^ 1#, and *p65*^–/–^ 2# 3D4/21 and PK15 cells were seeded in 12-well plates at a density of 1 × 10^5^ per well. On the next day, cells were treated as in **C**. The medium was then harvested and IL-1β **(E)** and IL-18 **(F)** secretion was quantified by ELISA. **P < 0.01, ***P < 0.001 determined by two-tailed Student’s *t*-test. **(G)** WT and *Sting*^–/–^ 3D4/21 cells were seeded in 60-mm dishes at a density of 4 × 10^5^ per dish. On the next day, cells were treated as in **C**. The medium was harvested to analyze mature IL-1β (P17) secretion, and the cells were harvested to analyze pro-IL-1β by immunoblotting analysis. **(H)** WT and *Sting*^–/–^ 3D4/21 cells were seeded in 12-well plates at a density of 1 × 10^5^ per well. On the next day, *Sting*^–/–^ 3D4/21 cells were transfected with plasmid for expression of STING-Flag plasmid (4 μg) for 24 h. Then, cells were treated as in **A**. The medium was then harvested and IL-1β and IL-18 secretion was quantified by ELISA. **P < 0.01, ***P < 0.001 determined by two-tailed Student’s *t*-test.

We asked whether G10 might promote porcine IL-1β and IL-18 secretion. Stimulation of human THP-1 cells with G10 did not affect IL-1β secretion (Supplementary Figure S1D). As a positive control, incubation of LPS-primed THP-1 cells with nigericin (LPS + Nig) significantly induced IL-1β secretion (Supplementary Figure S1D). However, G10 treatment stimulated STING-dependent secretion of IL-1β and IL-18 in 3D4/21 and PK15 cells (Figures 4A,B). IL-1β and IL-18 secretion was unchanged in WT and *Sting*^–/–^ 3D4/21 and PK15 cells treated with LPS + Nig (Figures 4A,B).

To further establish the role of G10 in IL-1β and IL-18 secretion in porcine cells, we assessed the secretion of these cytokines in cells with Sp1 or P65 expression interference. Knockdown of Sp1 by RNA interference decreased G10-induced IL-1β and IL-18 secretion in 3D4/21 and PK15 cells (Figures 4C,D). IL-1β and IL-18 secretion was constitutively up-regulated in response to LPS + Nig treatment, regardless of Sp1 deficiency (Figures 4C,D). In addition, P65-deficient 3D4/21 and PK15 cells, compared with WT cells, did not respond to G10 or to LPS + Nig in terms of induction of IL-1β and IL-18 secretion (Figures 4E,F). G10 promoted mature IL-1β secretion in 3D4/21 cells in a STING-dependent manner, as indicated by immunoblotting analysis of mature IL-1β (Figure 4G). Transfection of STING-Flag plasmid into *Sting*-deficient 3D4/21 cells resulted in G10-induced IL-1β and IL-18 secretion, to levels comparable to those in WT cells (Figure 4H). Together, these data demonstrate that STING-mediated NF-κB activation is essential for G10-induced IL-1β and IL-18 secretion in porcine cells.

### G10-Induced ASC Oligomerization and Caspase-1 Activation Promote IL-1β and IL-18 Maturation in Porcine Cells

**FIGURE 5:**
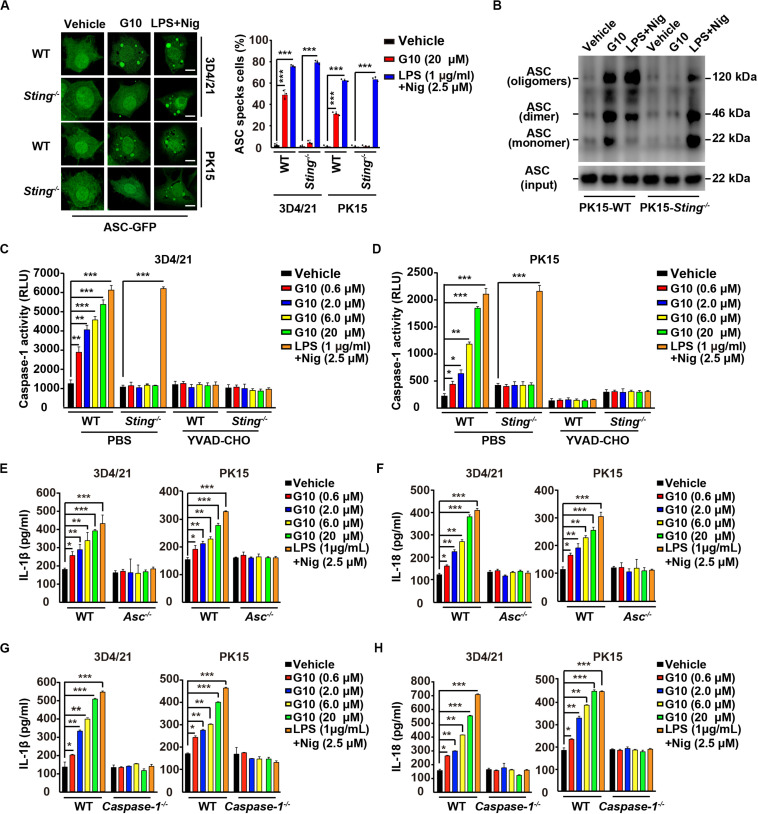
G10 induces ASC oligomerization and Caspase-1 activation in porcine cells. **(A)** WT and *Sting*^–/–^ 3D4/21 and PK15 cells were seeded in 12-well plates with coverslips at a density of 1 × 10^5^ per well. On the next day, cells were transfected with plasmid for expression of ASC-GFP (2 μg) for 24 h. Then, cells were treated with vehicle (DMSO), G10, and LPS + Nig at the indicated concentrations for 24 h. ASC oligomerization was assessed by fluorescence microscopy. Quantification of cells with ASC specks is shown on the right (*n* = 30 cells). Scale bar, 10 μm. ***P < 0.001 determined by two-tailed Student’s *t*-test. **(B)** WT and *Sting*^–/–^ PK15 cells were seeded in 60-mm dishes at a density of 4 × 10^5^ per dish. On the next day, cells were treated as in A. ASC oligomerization was assessed by immunoblotting analysis. **(C,D)** WT and *Sting*^–/–^ 3D4/21 and PK15 cells were seeded in 12-well plates at a density of 1 × 10^5^ per well. On the next day, cells were treated with vehicle (DMSO), G10, and LPS + Nig at the indicated concentrations in the absence (PBS) or presence of Caspase-1 inhibitor YVAD-CHO (5 μM) for 24 h. Caspase-1 activity was assessed with a Caspase-Glo 1 Inflammasome Assay kit. *P < 0.05, **P < 0.01, ***P < 0.001 determined by two-tailed Student’s *t*-test. **(E,F)** WT and *Asc*^–/–^ 3D4/21 and PK15 cells were seeded in 12-well plates at a density of 1 × 10^5^ per well. On the next day, cells were treated with vehicle (DMSO), G10 and LPS + Nig at the indicated concentrations for 24 h. The medium was then harvested and IL-1β **(E)** and IL-18 **(F)** secretion were quantified by ELISA. *P < 0.05, **P < 0.01, ***P < 0.001 determined by two-tailed Student’s *t*-test. **(G,H)** WT and *Caspase-1*^–/–^ 3D4/21 and PK15 cells were seeded in 12-well plates at a density of 1 × 10^5^ per well. On the next day, cells were treated as in **E**. The medium was then harvested and IL-1β **(G)** and IL-18 **(H)** secretion were quantified by ELISA. *P < 0.05, **P < 0.01, ***P < 0.001 determined by two-tailed Student’s *t*-test.

IL-1β and IL-18 maturation requires nucleation of the adaptor protein ASC, which controls Caspase-1 activation and subsequent cleavage of pro–IL-1β and pro–IL-18 (37). We observed that G10 treatment induced bright fluorescent ASC specks in WT but not *Sting*^–/–^ 3D4/21 and PK15 cells, whereas induction of ASC specks by LPS + Nig treatment was independent of STING (Figure 5A). Moreover, STING was responsible for G10-mediated induction of ASC oligomerization in PK15 cells, as indicated by immunoblotting analysis (Figure 5B).

Next, we investigated whether Caspase-1 might be activated by G10 treatment. As shown in Figures 5C,D, G10 dramatically increased Caspase-1 activity in 3D4/21 and in PK15 cells through STING. An inhibitor of Caspase-1, YVAD-CHO, blocked G10- and LPS + Nig-induced Caspase-1 activation (Figures 5C,D). Additionally, we examined the roles of ASC and Caspase-1 in G10-induced IL-1β and IL-18 secretion in *Asc*^–/–^ and *Caspase-1*^–/–^ 3D4/21 and PK15 cells generated by CRISPR/Cas9 editing (Supplementary Figures S1E,F). Knockout of ASC and Caspase-1 in 3D4/21 and PK15 cells abolished IL-1β and IL-18 secretion when cells were treated with G10 or LPS + Nig (Figures 5E–H). These data suggest that the STING/ASC/Caspase-1 axis is essential for G10-induced IL-1β and IL-18 secretion in porcine cells.

### G10 Activates the NLRP3 Inflammasome in Porcine Cells

**FIGURE 6:**
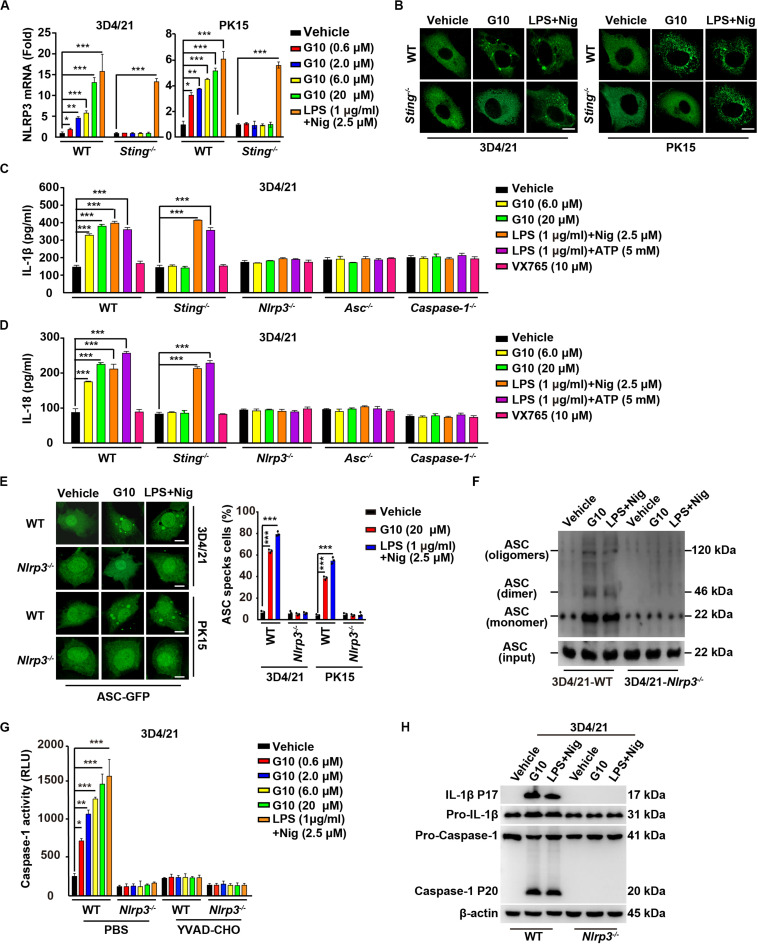
G10 activates the NLRP3 inflammasome in porcine cells. **(A)** WT and *Sting*^–/–^ 3D4/21 and PK15 cells were seeded in 12-well plates at a density of 1 × 10^5^ per well. On the next day, cells were treated with vehicle (DMSO), G10, and LPS + Nig at the indicated concentrations for 24 h. Total mRNA was then reverse-transcribed to cDNA and NLRP3 mRNA was assessed by RT-qPCR analysis. The results were normalized to the level of β-actin expression. *P < 0.05, **P < 0.01, ***P < 0.001 determined by two-tailed Student’s *t*-test. **(B)** WT and *Sting*^–/–^ 3D4/21 and PK15 cells were seeded in 12-well plates with coverslips at a density of 1 × 10^5^ per well. On the next day, cells were transfected with plasmid for expression of NLRP3-Flag (2 μg) for 24 h. Then, cells were treated with vehicle (DMSO), G10 (20 μM), and LPS + Nig (1 μg/ml + 2.5 μM) for 24 h. NLRP3 activation was assessed by immunofluorescence analysis with antibody against Flag. Scale bar, 10 μm. **(C,D)** WT, *Sting*^–/–^, *Nlrp3*^–/–^, *Asc*^–/–^, and *Caspase-1*^–/–^ 3D4/21 cells were seeded in 12-well plates at a density of 1 × 10^5^ per well. On the next day, cells were treated with vehicle (DMSO), G10, LPS + Nig, LPS + ATP, and VX765 at the indicated concentrations for 24 h. The medium was then harvested and IL-1β **(C)** and IL-18 **(D)** secretion were quantified by ELISA. ***P < 0.001 determined by two-tailed Student’s *t*-test. **(E)** WT and *Nlrp3*^–/–^ 3D4/21 and PK15 cells were seeded in 12-well plates with coverslips at a density of 1 × 10^5^ per well. On the next day, cells were transfected with plasmid for expression of ASC-GFP (2 μg) for 24 h. Then, cells were treated as in **B**. ASC oligomerization was assessed by fluorescence microscopy. Quantification of cells with ASC specks is shown on the right (*n* = 30 cells). Scale bar, 10 μm. ***P < 0.001 determined by two-tailed Student’s *t*-test. **(F)** WT and *Nlrp3*^–/–^ 3D4/21 cells were seeded in 60-mm dishes at a density of 4 × 10^5^ per dish. On the next day, cells were treated as in **B**. ASC oligomerization was assessed by immunoblotting analysis. **(G)** WT and *Nlrp3*^–/–^ 3D4/21 cells were seeded in 12-well plates at a density of 1 × 10^5^ per well. On the next day, cells were treated with vehicle (DMSO), G10, and LPS + Nig at the indicated concentrations in the absence (PBS) or presence of Caspase-1 inhibitor YVAD-CHO (5 μM) for 24 h. Caspase-1 activity was assessed with a Caspase-Glo 1 Inflammasome Assay kit. *P < 0.05, **P < 0.01, ***P < 0.001 determined by two-tailed Student’s *t*-test. **(H)** WT and *Nlrp3*^–/–^ 3D4/21 cells were seeded in 60-mm dishes at a density of 4 × 10^5^ per dish. On the next day, cells were treated as in **B**. The medium was harvested to analyze mature IL-1β (P17) secretion, and the cells were harvested to analyze pro-IL-1β, pro-Caspase-1, and cleaved Caspase-1 (P20) by immunoblotting analysis.

Because our data suggested that G10 is a bona fide inflammasome activator, we sought to investigate which PRRs might be involved in G10-mediated activation of inflammasomes. We first analyzed the mRNA expression of inflammasome effectors under G10 treatment. As indicated by RT-qPCR analysis, G10 did not induce NLRP1 transcription in 3D4/21 and PK15 cells (Supplementary Figures S2A,B). However, NLRP3 mRNA was significantly up-regulated by G10 treatment in WT but not in *Sting*^–/–^ 3D4/21 and PK15 cells (Figure 6A). Because NLRP3 inflammasome activation leads to NLRP3 aggregation (38), we detected NLRP3 aggregation by immunofluorescence. We observed that multiple puncta of NLRP3 appeared after G10 treatment in WT but not STING-deficient 3D4/21 and PK15 cells (Figure 6B). We stimulated 3D4/21 cells with G10 to observe whether STING co-localized to NLRP3. No obvious co-localization was observed when cells were treated with DMSO (Supplementary Figure S2C). G10 treatment induced multiple puncta of STING formation, some of which co-localized with NLRP3 (Supplementary Figure S2C). These results demonstrated that G10 activates porcine NLRP3.

We then established the roles of NLRP3 in IL-1β and IL-18 secretion, ASC oligomerization, and Caspase-1 activation in response to G10 treatment. After G10 treatment, knockout of STING, NLRP3, ASC, and Caspase-1 in 3D4/21 and PK15 cells suppressed IL-1β and IL-18 secretion (Supplementary Figure S2D and Figures 6C,D). Treatment of WT and *Sting*^–/–^ 3D4/21 and PK15 cells with LPS + Nig or LPS + ATP [another canonical NLRP3 activator (24)] stimulated IL-1β and IL-18 secretion (Figures 6C,D). An inhibitor of Caspase-1, VX756 (39), prevented WT, *Sting*^–/–^, *Nlrp3*^–/–^, and *Asc*^–/–^ 3D4/21 and PK15 cells from secreting IL-1β and IL-18 in response to G10, LPS + Nig, or LPS + ATP (Figures 6C,D). ASC oligomerization disappeared in G10- and LPS + Nig-treated *Nlrp3*^–/–^ cells, as indicated by fluorescence analysis of ASC specks (Figure 6E) or by immunoblotting analysis of ASC oligomerization (Figure 6F). Caspase-1 was not activated in *Nlrp3*^–/–^ 3D4/21 cells treated with G10 or LPS + Nig (Figure 6G). The cleavage of pro-IL-1β and pro-Caspase-1 induced by G10 and LPS + Nig did not occur, owing to the NLRP3 ablation in 3D4/21 cells (Figure 6H). Together, these data indicate that G10 promotes canonical NLRP3 inflammasome activation in porcine cells.

### G10 Induces Potassium Efflux, Thus Activating the NLRP3 Inflammasome in Porcine Cells

**FIGURE 7:**
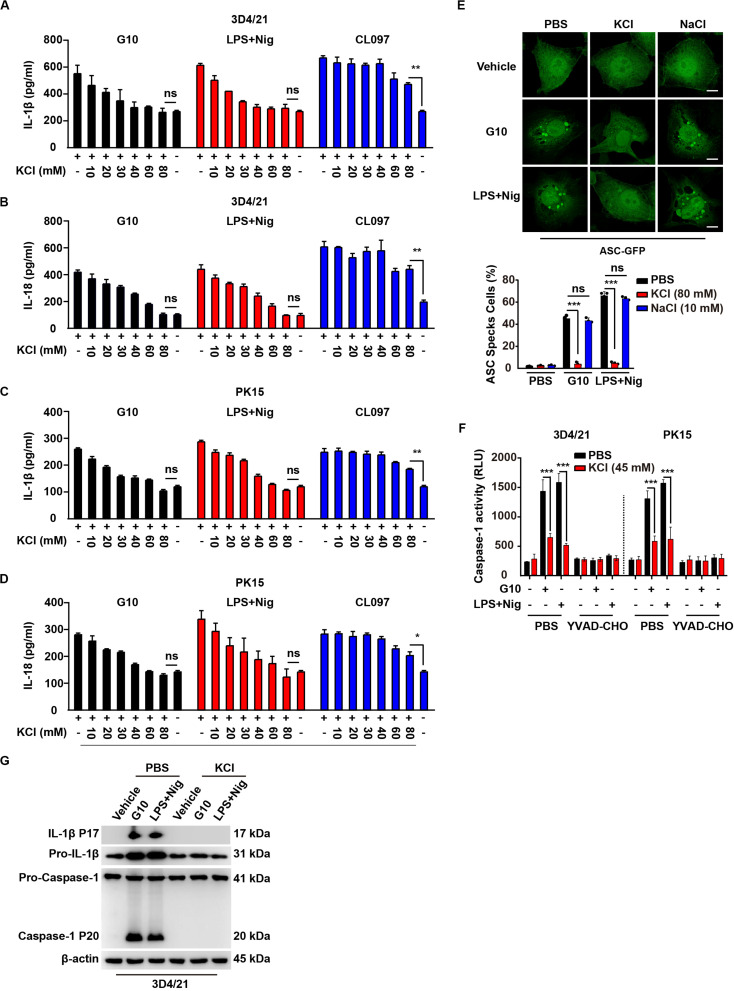
G10-mediated activation of the NLRP3 inflammasome requires potassium flux in porcine cells. **(A–D)** 3D4/21 **(A,B)** and PK15 **(C,D)** cells were seeded in 12-well plates at a density of 1 × 10^5^ per well. On the next day, cells were treated with G10 (20 μm), LPS + Nig (1 μg/ml + 2.5 μM), and CL097 (70 μM) in the absence or presence of KCl (10–80 mM) for 24 h. The medium was then harvested and IL-1β **(A,C)** and IL-18 **(B,D)** secretion were quantified by ELISA. *P < 0.05, **P < 0.01 determined by two-tailed Student’s *t*-test. ns, no significance. **(E)** 3D4/21 cells were seeded in 12-well plates with coverslips at a density of 1 × 10^5^ per well. On the next day, cells were transfected with plasmid for expression of ASC-GFP (2 μg) for 24 h. Then, cells were treated with vehicle (DMSO), G10 (20 μm), and LPS + Nig (1 μg/ml + 2.5 μM) in the absence (PBS) or presence of KCl (80 mM) and NaCl (10 mM) for 24 h. ASC oligomerization was assessed by fluorescence microscopy. Quantification of cells with ASC specks is shown at the bottom (*n* = 30 cells). Scale bar, 10 μm. ***P < 0.001 determined by two-tailed Student’s *t*-test. ns, no significance. **(F)** 3D4/21 and PK15 cells were seeded in 12-well plates at a density of 1 × 10^5^ per well. On the next day, cells were treated with G10 (20 μm) and LPS + Nig (1 μg/ml + 2.5 μM) as indicated in the absence (PBS) or presence of KCl (45 mM) and Caspase-1 inhibitor YVAD-CHO (5 μM) for 24 h. Caspase-1 activity was assessed with a Caspase-Glo 1 Inflammasome Assay kit. ***P < 0.001 determined by two-tailed Student’s *t*-test. **(G)** 3D4/21 cells were seeded in 60-mm dishes at a density of 4 × 10^5^ per dish. On the next day, cells were treated with vehicle (DMSO), G10 (20 μm), and LPS + Nig (1 μg/ml + 2.5 μM) in the absence (PBS) or presence of KCl (80 mM) for 24 h. The medium was harvested to analyze mature IL-1β (P17) secretion, and the cells were harvested to analyze pro-IL-1β, pro-Caspase-1 and cleaved Caspase-1 (P20) by immunoblotting analysis.

Several mechanisms have been proposed to be critical for NLRP3 inflammasome activation, such as potassium efflux, calcium mobilization, mitochondrial damage, and ROS (21–24). We sought to address the mechanism through which G10 activates the NLRP3 inflammasome. Because potassium efflux has been proposed to play a central role in NLRP3 inflammasome activation (40), we tested whether K^+^ replenishment might rescue the effects on IL-1β and IL-18 secretion induced by G10. ELISA of IL-1β and IL-18 indicated that supplementation with K^+^ gradually down-regulated G10-induced secretion of IL-1β and IL-18 from 3D4/21 and PK15 cells to basal levels (Figures 7A–D). Nigericin is an ionophore that induces potassium efflux, thus promoting NLRP3 inflammasome activation (40), and K^+^ replenishment also down-regulated LPS + Nig-induced IL-1β and IL-18 secretion to the levels observed in untreated cells (Figures 7A–D). NLRP3 inflammasome activation by CL097 is K^+^ efflux-independent (41). Extracellular K^+^ did not completely abrogate CL097-stimulated IL-1β and IL-18 secretion, as expected (Figures 7A–D).

G10 and LPS + Nig induced formation of ASC specks in 3D4/21 cells, and this effect was reversed by K^+^ replenishment but not Na^+^ replenishment (Figure 7E). Extracellular K^+^ inhibited Caspase-1 activation in 3D4/21 and PK15 cells under G10 or LPS + Nig treatment (Figure 7F). K^+^ replenishment inhibited G10- and LPS + Nig-induced cleavage of pro-IL-1β and pro-Caspase-1 in 3D4/21 cells (Figure 7G). Together, these results suggest that G10 activates the NLRP3 inflammasome through K^+^ efflux in porcine cells.

### Inhibition of the NLRP3 Inflammasome Enhances G10-Induced Type I IFN in Porcine Cells

**FIGURE 8:**
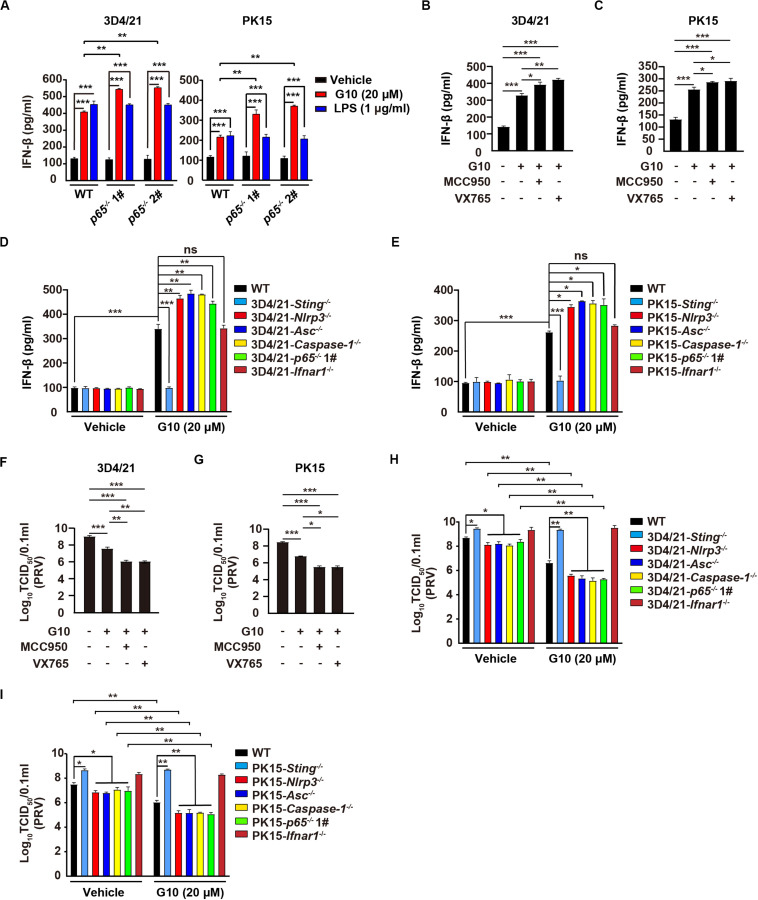
Inhibition of the NLRP3 inflammasome augments G10-induced type I IFN and antiviral activity in porcine cells. **(A)** WT, *p65*^–/–^ 1#, and *p65*^–/–^ 2# 3D4/21 and PK15 cells were seeded in 12-well plates at a density of 1 × 10^5^ per well. On the next day, cells were treated with vehicle (DMSO), G10 (20 μM), and LPS (1 μg/ml) for 24 h. The medium was then harvested and IFN-β secretion was quantified by ELISA. **P < 0.01, ***P < 0.001 determined by one-way ANOVA. **(B,C)** 3D4/21 **(B)** and PK15 **(C)** cells were seeded in 12-well plates at a density of 1 × 10^5^ per well. On the next day, cells were treated with G10 (20 μM), MCC950 (10 μM), and VX765 (10 μM) as indicated for 24 h. The medium was then harvested and IFN-β secretion was quantified by ELISA. *P < 0.05, **P < 0.01, ***P < 0.001 determined by one-way ANOVA. **(D,E)** WT, *Sting*^–/–^, *Nlrp3*^–/–^, *Asc*^–/–^, *Caspase-1*^–/–^, *p65*^–/–^ 1#, and *Ifnar1*^–/–^ 3D4/21 **(D)** and WT, *Sting*^–/–^, *Nlrp3*^–/–^, *Asc*^–/–^, *Caspase-1*^–/–^, *p65*^–/–^ 1#, and *Ifnar1*^–/–^ PK15 **(E)** cells were seeded in 12-well plates at a density of 1 × 10^5^ per well. On the next day, cells were treated with vehicle (DMSO) or G10 (20 μM) for 24 h. The medium was then harvested and IFN-β secretion was quantified by ELISA. *P < 0.05, **P < 0.01, ***P < 0.001 determined by one-way ANOVA. ns, no significance. **(F,G)** 3D4/21 **(F)** and PK15 **(G)** cells were seeded in 12-well plates at a density of 1 × 10^5^ per well. On the next day, cells were infected with PRV-QXX (MOI = 1) and simultaneously treated as in **B**. Virus was harvested by three freeze–thaw cycles and PRV titer was assessed with TCID_50_ assays. *P < 0.05, **P < 0.01, ***P < 0.001 determined by one-way ANOVA. **(H,I)** WT, *Sting*^–/–^, *Nlrp3*^–/–^, *Asc*^–/–^, *Caspase-1*^–/–^, *p65*^–/–^ 1#, and *Ifnar1*^–/–^ 3D4/21 **(H)** and WT, *Sting*^–/–^, *Nlrp3*^–/–^, *Asc*^–/–^, *Caspase-1*^–/–^, *p65*^–/–^ 1#, and *Ifnar1*^–/–^ PK15 **(I)** cells were seeded in 12-well plates at a density of 1 × 10^5^ per well. On the next day, cells were treated as in **D**. Virus was harvested by three freeze–thaw cycles and PRV titer was assessed with TCID_50_ assays. *P < 0.05, **P < 0.01 determined by one-way ANOVA.

Type I IFN and inflammasomes are reciprocally regulated, in a process essential for immune homeostasis (42). Therefore, we defined the role of the NLRP3 inflammasome in the regulation of type I IFN in response to G10 in porcine cells. Ablation of *p65* further increased G10-, but not LPS-induced IFN-β and ISG15 mRNA transcription, as well as IFN-β secretion in 3D4/21 and PK15 cells (Supplementary Figures S3A,B and Figure 8A). Inhibition of the NLRP3 inflammasome by MCC950, or of Caspase-1 by VX765, prevented G10-mediated induction of IL-1β and IL18 secretion and enhanced IFN-β secretion in 3D4/21 and PK15 cells (Supplementary Figures S3C,D and Figures 8B,C). In addition, G10-induced *Nlrp3*^–/–^, *Asc*^–/–^, *Caspase-1*^–/–^, and *p65*^–/–^ 3D4/21 and PK15 cells generated more IFN-β than WT and *Ifnar1*^–/–^ cells (Figures 8D,E). These data demonstrate that inhibition of the NLRP3 inflammasome enhances G10-induced type I IFN expression in porcine cells.

We then asked whether G10 might effectively prevent PRV infection through inhibition of the NLRP3 inflammasome in porcine cells. Viral titer assays indicated that G10 combined with MCC950 or VX765 had greater antiviral activity against PRV than G10 alone in 3D4/21 and PK15 cells, but not in *Ifnar1*^–/–^ 3D4/21 cells (Figures 8F,G and Supplementary Figures S3E,F). Knockout of *Sting* or *Ifnar1* promoted PRV proliferation, whereas knockout of *Nlrp3*, *Asc*, *Caspase-1*, and *p65* inhibited PRV proliferation (Figures 8H,I). Compared with WT 3D4/21 and PK15 cells, *Nlrp3*^–/–^, *Asc*^–/–^, *Caspase-1*^–/–^, and *p65*^–/–^ cells treated with G10 showed significantly repressed PRV proliferation (Figures 8H,I). Collectively, these data indicate that G10-induced NLRP3 inflammasome activation negatively regulates type I IFN in porcine cells.

## Discussion

**FIGURE 9:**
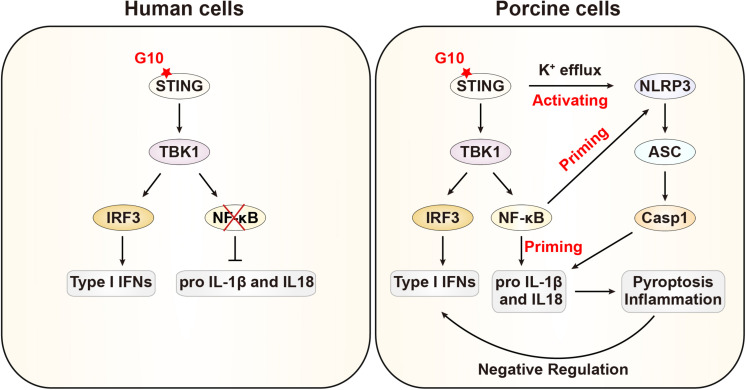
Schematic model showing G10 activation of the NLRP3 inflammasome in porcine cells. In human cells, G10 activates only type I IFN. In porcine cells, G10 also activates the NF-κB signaling pathway, which is a priming signal for NLRP3 inflammasome activation. G10 induces potassium efflux and triggers NLRP3 inflammasome activation, which negatively regulates type I IFN.

In this study, we demonstrated that G10 activates the porcine type I IFN response, in line with previous observations in human cells. Intriguingly, we further demonstrate that G10 primes the NLRP3 inflammasome through up-regulation of NLRP3, pro–IL-1β, and pro–IL-18 through STING, in contrast to the process in human cells. Furthermore, G10 induces potassium efflux and the oligomerization of NLRP3 and ASC, thus resulting in the formation of active Caspase-1, which in turn induces the secretion of mature IL-1β and IL-18. Treatment of NLRP3 inflammasome-deficient cells with G10 enhanced type I IFN expression and effectively inhibited viral infection. Our data indicate that type I IFN is negatively regulated by the NLRP3 inflammasome in porcine cells (Figure 9).

Nuclear factor-κB, a central mediator of immune and stress responses, is activated by multiple intra- and extracellular stimuli (43, 44). Our work shows that G10 can activate the NF-κB pathway in porcine cells, but this activation does not occur in human cells. We found that G10 induces porcine *p65* transcription through STING and Sp1. A previous study has indicated that the human *p65* promoter lacks both TATA and CCAAT consensus sequences and contains three consensus binding sites for Sp1 (45). Human cytomegalovirus (HCMV) infection increases Sp1 expression and activates transcription of *p65* through its promoter Sp1-binding sites (46, 47). Because cGAS and STING are key sensors of HCMV in primary human monocyte-derived DCs and macrophages (48, 49), our data may explain the mechanism through which HCMV infection activates *p65* transcription. We also show that LPS-induced *p65* transcription is independent of STING and Sp1 in porcine cells. LPS activates NF-κB through Toll-like receptor 4-mediated signaling and causes Sp1 protein degradation by an LPS-inducible Sp1-degrading enzyme (50). Therefore, these findings combined with our current findings suggest that NF-κB is regulated by diverse stimuli through distinct mechanisms in different species.

Several studies have suggested that STING is involved in inflammasome activation. The cyclic dinucleotides 3′5′-diadenylate and 3′5′-diguanylate are bacterial second messengers that activate STING-mediated innate immune responses (51). They stimulate robust secretion of IL-1β through NLRP3 inflammasome signaling that is independent of STING (52). The intrinsic STING agonist cGAMP is catalyzed by cGAS from GTP and ATP (53), and it induces inflammasome activation through a STING, AIM2, NLRP3, ASC, and Caspase-1 dependent process in human and mouse cells (54). Cyclic dinucleotides from prokaryotes and eukaryotes are involved in different pathways of STING-mediated activation of inflammasomes. Nevertheless, our data demonstrate that STING is essential for G10-mediated activation of the canonical NLRP3 inflammasome in porcine cells. We did not determine whether AIM2 is involved in G10-induced NLRP3 inflammasome activation, because AIM2 is not present in pigs (55). Importantly, given that STING is a promising target for cancer immunotherapy, our results suggest that the roles of STING agonist-induced type I IFN in inflammasome activation should be assessed in clinical trials.

The sustained robust inflammation may lead to collateral damage due to the overproduction of inflammatory cytokines (56). Aside from the antimicrobial functions of IFNs, aberrant IFN production is associated with a variety of autoimmune disorders (57). We demonstrated that the NLRP3 inflammasome negatively regulates type I IFN, which may be essential for immune homeostasis in pigs. Multiple reports have demonstrated that the NLRP3 inflammasome can induce and regulate the development of adaptive immunity (58). Both IL-1β and IL-1β are involved in T cell activation and memory cell formation (59–62). Additionally, IL-1β and IL-18 have adjuvant capacity. Moreover, IL-1β enhances humoral immunity (63), and IL-18 augments IgE antibody production (64). Therefore, these beneficial aspects on the NLRP3 inflammasome may support the use of G10 as an effective vaccine adjuvant in the pig industry.

## Conclusion

Stimulator of interferon genes is an ER-resident transmembrane protein that integrates cytosolic dsDNA-triggered activation of innate immune responses. G10 is a synthetic agonist of human STING that stimulates only STING-dependent IFN expression. However, we found that G10 acts on STING and activates both type I IFN and the canonic NLRP3 inflammasome. G10 activates the NLRP3 inflammasome in porcine cells through simultaneous priming and activation. Our data indicate that the G10-activated NLRP3 inflammasome negatively regulates type I IFN, a process that may be essential for immune homeostasis in pigs.

## Data Availability Statement

All datasets presented in this study are included in the article/Supplementary Material.

## Author Contributions

S-LM, LZ, Y-KG, G-YY, JW, and B-BC: project conception and design. S-LM, LZ, Y-KG, SZ, GL-L, Y-XM, YY-Z, W-RC, and LY: data collection. S-LM, LZ, Y-KG, SZ, and G-LL: data analysis and interpretation. S-LM, G-YY, JW, and B-BC: original manuscript drafting. JW and B-BC: manuscript review, editing, and final approval. All authors contributed to the article and approved the submitted version.

## Conflict of Interest

The authors declare that the research was conducted in the absence of any commercial or financial relationships that could be construed as a potential conflict of interest.

## Supplementary Materials

FIGURE S1 G10 activates porcine NF-κB signaling pathway. **(A)** WT and *Sting*^–/–^ 3D4/21 cells were seeded in 12-well plates at a density of 1 × 10^5^ per well. On the next day, *Sting*^–/–^ 3D4/21 cells were transfected with plasmid for expression of STING-Flag (4 μg) for 24 h. Then cells were treated with vehicle (DMSO), G10 and LPS + Nig at the indicated concentrations for 24 h. Total mRNA was then reverse-transcribed to cDNA and P65 mRNA was assessed by RT-qPCR analysis. The results were normalized to the level of β-actin expression. **P* < 0.05, ***P* < 0.01, ****P* < 0.001 determined by two-tailed Student’s *t*-test. **(B)** THP-1 cells were seeded and differentiated in 12-well plates with coverslips at a density of 2 × 10^5^ per well. On the next day, cells were treated with DMSO, G10 (20 μM) and LPS (1 μg/ml) for 24 h. Translocation of P65 into the nucleus (DAPI) was assessed by immunofluorescence analysis with antibody against P65. Quantification of cells with nuclear localized P65 is shown on the right (*n* = 30 cells). Scale bar, 10 μm. ****P* < 0.001 determined by two-tailed Student’s *t*-test. ns, no significance. **(C)** Schematic representation of the porcine *p65* genomic structure and DNA sequencing results for the indicated knockout cells. Protospacer sequence is shown in red. The PAM sequence is framed by black boxes. **(D)** THP-1 cells were seeded and differentiated in 12-well plates with coverslips at a density of 2 × 10^5^ per well. On the next day, cells were treated as in **A**. The medium was then harvested and IL-1β secretion was quantified by ELISA. ****P* < 0.001 determined by two-tailed Student’s *t*-test. **(E,F)** Schematic representations of the porcine *Asc* **(E)** and *Caspase-1* **(F)** genomic structure and DNA sequencing results for the indicated knockout cells. Protospacer sequences are shown in red. The PAM sequences are framed by black boxes.

FIGURE S2 G10 activates porcine NLRP3 inflammasome. **(A,B)** WT, *Sting*^–/–^ 3D4/21 **(A)** and WT, *Sting*^–/–^ PK15 **(B)** cells were seeded in 12-well plates at a density of 1 × 10^5^ per well. On the next day, cells were treated with vehicle (DMSO), G10 and LPS + Nig at the indicated concentrations for 24 h. Total mRNA was then reverse-transcribed to cDNA and NLRP1 mRNA was assessed by RT-qPCR analysis. The results were normalized to the level of β-actin expression. **(C)** 3D4/21 cells were seeded in 12-well plates with coverslips at a density of 1 × 10^5^ per well. On the next day, cells were transfected with plasmid for expression of NLRP3-Flag (2 μg) for 24 h. Then cells were treated with DMSO and G10 (20 μM) for 24 h. Co-localization of STING and NLRP3 was assessed by immunofluorescence analysis with antibodies against STING and Flag. Scale bar, 10 μm. **(D)** Schematic representations of the porcine *Nlrp3* genomic structure and DNA sequencing results of *Nlrp3*^–/–^ 3D4/21 and PK15 cells. The protospacer sequence is shown in red. The PAM sequence is framed by a black box.

FIGURE S3 Inhibition of inflammasome activation enhances G10-induce type I IFN response. **(A,B)** WT, *p65*^–/–^ 1#, *p65*^–/–^ 2# 3D4/21, and PK15 cells were seeded in 12-well plates at a density of 1 × 10^5^ per well. On the next day, cells were treated with vehicle (DMSO), G10 (20 μM) and LPS (1 μg/ml) for 24 h. Total mRNA was then reverse-transcribed to cDNA and IFN-β **(A)** and ISG15 **(B)** mRNA was assessed by RT-qPCR analysis. The results were normalized to the level of β-actin expression. ***P* < 0.01, ****P* < 0.001 determined by two-tailed Student’s *t*-test. **(C)** 3D4/21 cells were seeded in 12-well plates at a density of 1 × 10^5^ per well. On the next day, cells were untreated or treated with G10 (20 μM), MCC950 (10 μM), and VX765 (10 μM) as indicated for 24 h. The medium was then harvested and IL-1β and IL-18 secretion was quantified by ELISA. ****P* < 0.001 determined by two-tailed Student’s *t*-test. ns, no significance. **(D)** PK15 cells were seeded in 12-well plates at a density of 1 × 10^5^ per well. On the next day, cells were treated as in **C**. The medium was then harvested and IL-1β and IL-18 secretion was quantified by ELISA. ***P* < 0.01 determined by two-tailed Student’s *t*-test. ns, no significance. **(E)** WT and *Ifnar1*^–/–^ 3D4/21 cells were seeded in 12-well plates at a density of 1 × 10^5^ per well. On the next day, cells were treated as in **C**. Virus was harvested by three freeze-thaw cycles and PRV titer was assessed with TCID_50_ assays. ***P* < 0.01, ****P* < 0.001 determined by one-way ANOVA. **(F)** PRV titer was assessed with plaque assays from **E**. **P* < 0.05, ***P* < 0.01, ****P* < 0.001 determined by one-way ANOVA.
