# Supplementary figures and images for: The Human-Specific STING Agonist G10 Activates Type I Interferon and the NLRP3 Inflammasome in Porcine Cells
Source: Front Immunol. 2020 Sep 24;11:575818. doi: 10.3389/fimmu.2020.575818 (PMC7543045; doi:10.3389/fimmu.2020.575818)

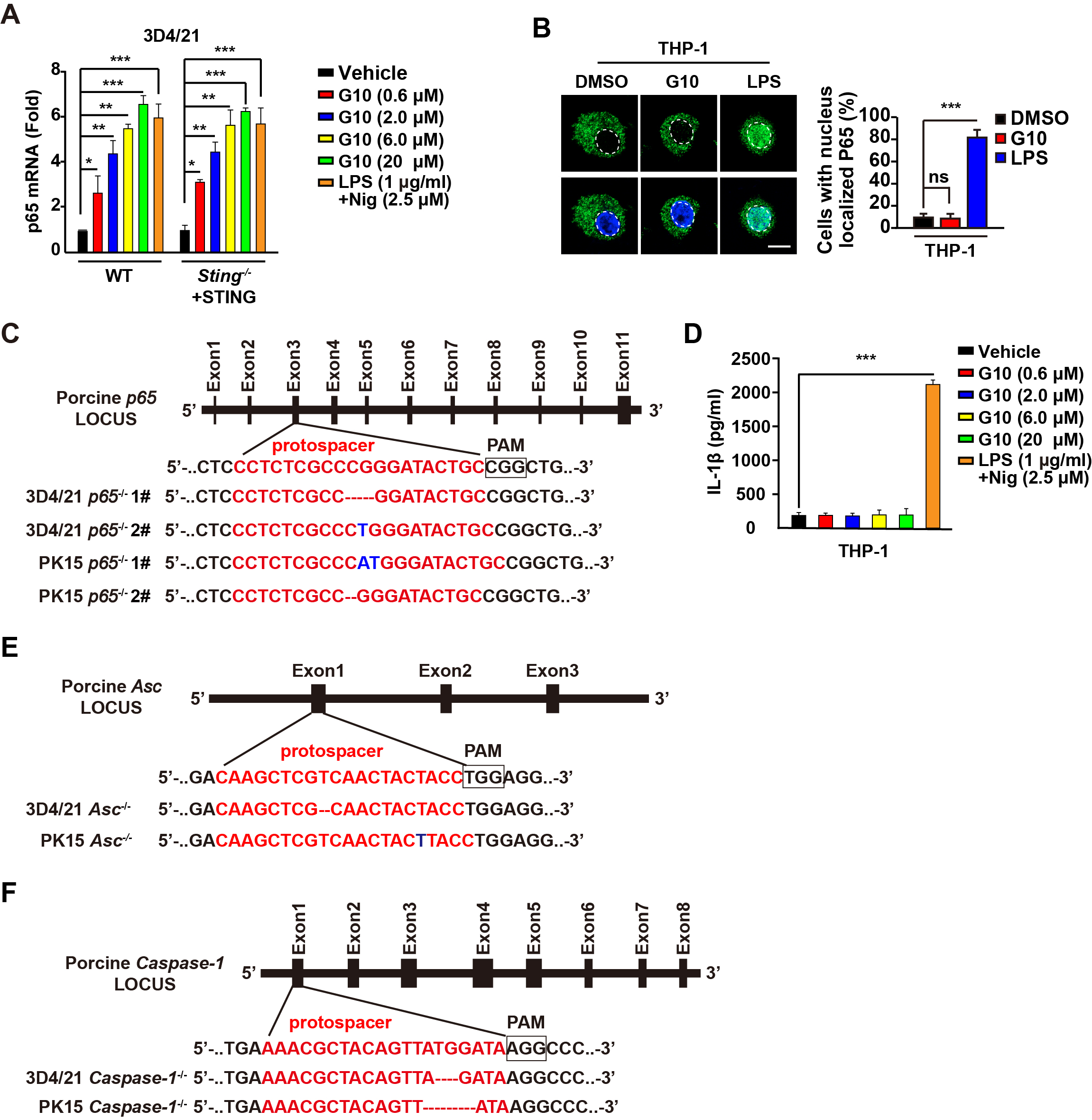

Supplement: FIGURE S1 — G10 activates porcine NF-κB signaling pathway. (A) WT and Sting–/– 3D4/21 cells were seeded in 12-well plates at a density of 1 × 10^5^ per well. On the next day, Sting–/– 3D4/21 cells were transfected with plasmid for expression of STING-Flag (4 μg) for 24 h. Then cells were treated with vehicle (DMSO), G10 and LPS + Nig at the indicated concentrations for 24 h. Total mRNA was then reverse-transcribed to cDNA and P65 mRNA was assessed by RT-qPCR analysis. The results were normalized to the level of β-actin expression. *P < 0.05, **P < 0.01, ***P < 0.001 determined by two-tailed Student’s t-test. (B) THP-1 cells were seeded and differentiated in 12-well plates with coverslips at a density of 2 × 10^5^ per well. On the next day, cells were treated with DMSO, G10 (20 μM) and LPS (1 μg/ml) for 24 h. Translocation of P65 into the nucleus (DAPI) was assessed by immunofluorescence analysis with antibody against P65. Quantification of cells with nuclear localized P65 is shown on the right (n = 30 cells). Scale bar, 10 μm. ***P < 0.001 determined by two-tailed Student’s t-test. ns, no significance. (C) Schematic representation of the porcine p65 genomic structure and DNA sequencing results for the indicated knockout cells. Protospacer sequence is shown in red. The PAM sequence is framed by black boxes. (D) THP-1 cells were seeded and differentiated in 12-well plates with coverslips at a density of 2 × 10^5^ per well. On the next day, cells were treated as in A. The medium was then harvested and IL-1β secretion was quantified by ELISA. ***P < 0.001 determined by two-tailed Student’s t-test. (E,F) Schematic representations of the porcine Asc (E) and Caspase-1 (F) genomic structure and DNA sequencing results for the indicated knockout cells. Protospacer sequences are shown in red. The PAM sequences are framed by black boxes. [file Image_1.jpeg]

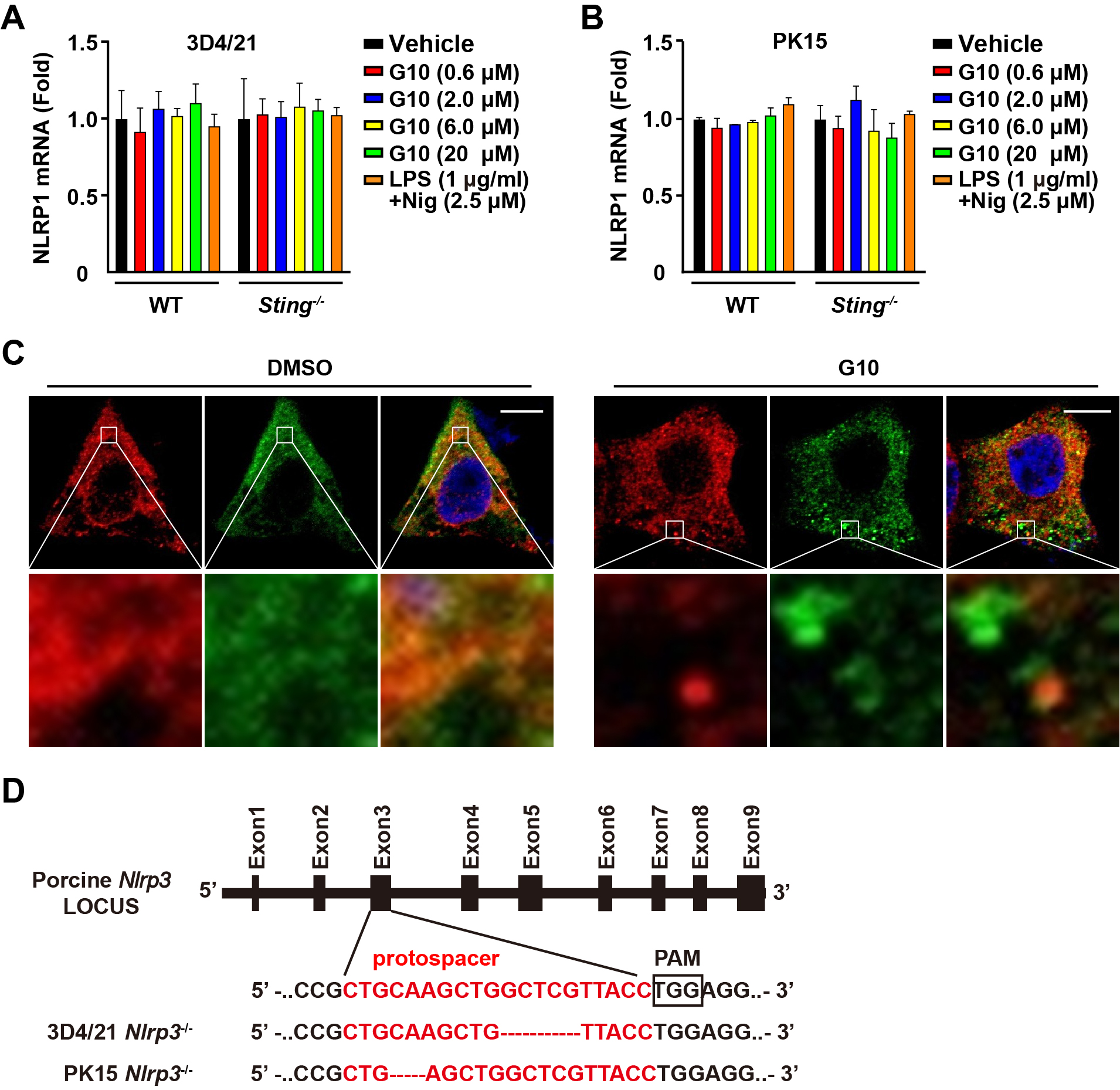

Supplement: FIGURE S2 — G10 activates porcine NLRP3 inflammasome. (A,B) WT, Sting–/– 3D4/21 (A) and WT, Sting–/– PK15 (B) cells were seeded in 12-well plates at a density of 1 × 10^5^ per well. On the next day, cells were treated with vehicle (DMSO), G10 and LPS + Nig at the indicated concentrations for 24 h. Total mRNA was then reverse-transcribed to cDNA and NLRP1 mRNA was assessed by RT-qPCR analysis. The results were normalized to the level of β-actin expression. (C) 3D4/21 cells were seeded in 12-well plates with coverslips at a density of 1 × 10^5^ per well. On the next day, cells were transfected with plasmid for expression of NLRP3-Flag (2 μg) for 24 h. Then cells were treated with DMSO and G10 (20 μM) for 24 h. Co-localization of STING and NLRP3 was assessed by immunofluorescence analysis with antibodies against STING and Flag. Scale bar, 10 μm. (D) Schematic representations of the porcine Nlrp3 genomic structure and DNA sequencing results of Nlrp3–/– 3D4/21 and PK15 cells. The protospacer sequence is shown in red. The PAM sequence is framed by a black box. [file Image_2.jpeg]

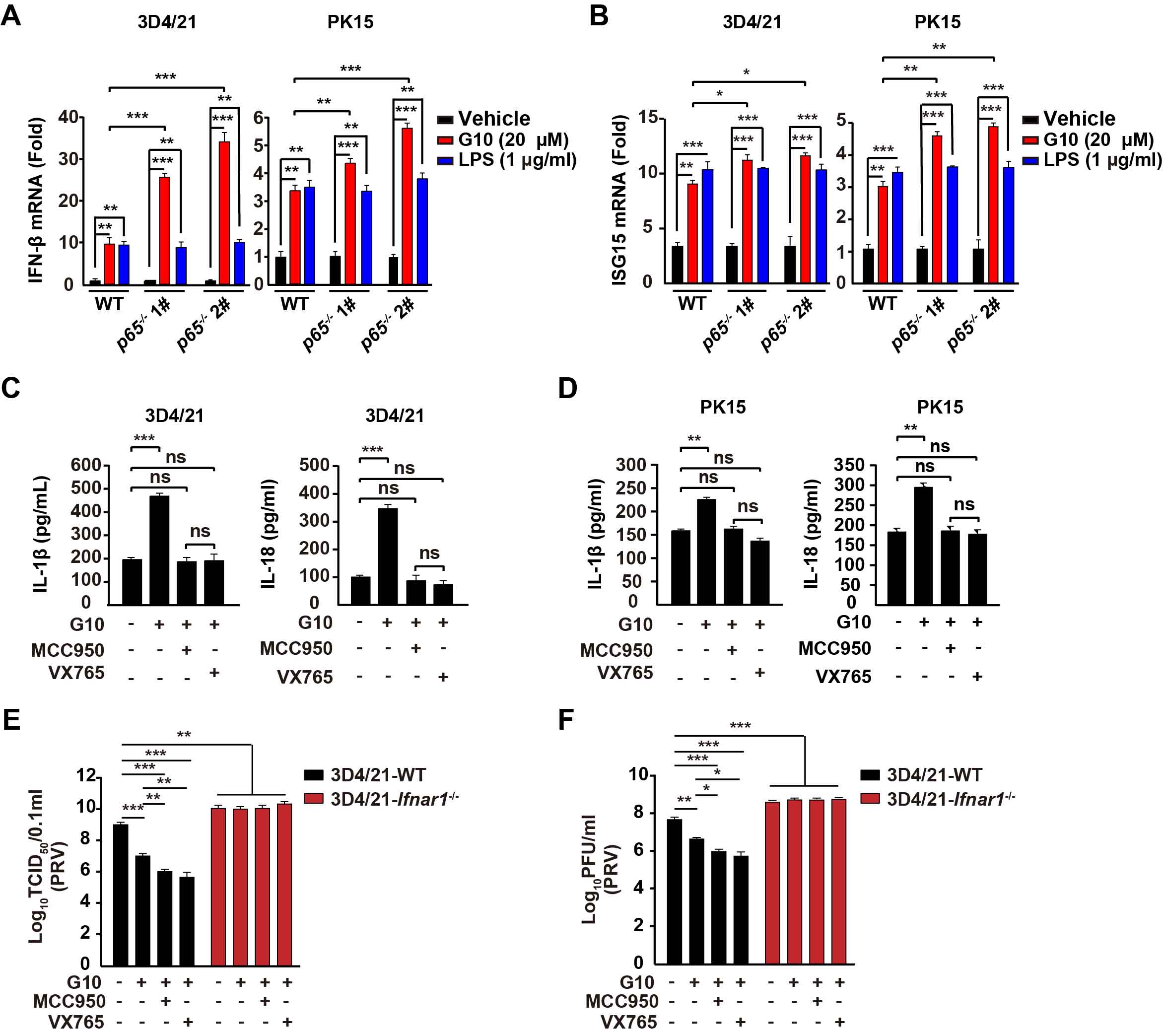

Supplement: FIGURE S3 — Inhibition of inflammasome activation enhances G10-induce type I IFN response. (A,B) WT, p65–/– 1#, p65–/– 2# 3D4/21, and PK15 cells were seeded in 12-well plates at a density of 1 × 10^5^ per well. On the next day, cells were treated with vehicle (DMSO), G10 (20 μM) and LPS (1 μg/ml) for 24 h. Total mRNA was then reverse-transcribed to cDNA and IFN-β (A) and ISG15 (B) mRNA was assessed by RT-qPCR analysis. The results were normalized to the level of β-actin expression. **P < 0.01, ***P < 0.001 determined by two-tailed Student’s t-test. (C) 3D4/21 cells were seeded in 12-well plates at a density of 1 × 10^5^ per well. On the next day, cells were untreated or treated with G10 (20 μM), MCC950 (10 μM), and VX765 (10 μM) as indicated for 24 h. The medium was then harvested and IL-1β and IL-18 secretion was quantified by ELISA. ***P < 0.001 determined by two-tailed Student’s t-test. ns, no significance. (D) PK15 cells were seeded in 12-well plates at a density of 1 × 10^5^ per well. On the next day, cells were treated as in C. The medium was then harvested and IL-1β and IL-18 secretion was quantified by ELISA. **P < 0.01 determined by two-tailed Student’s t-test. ns, no significance. (E) WT and Ifnar1–/– 3D4/21 cells were seeded in 12-well plates at a density of 1 × 10^5^ per well. On the next day, cells were treated as in C. Virus was harvested by three freeze-thaw cycles and PRV titer was assessed with TCID50 assays. **P < 0.01, ***P < 0.001 determined by one-way ANOVA. (F) PRV titer was assessed with plaque assays from E. *P < 0.05, **P < 0.01, ***P < 0.001 determined by one-way ANOVA. [file Image_3.jpeg]
